# Inhibition of *Pasteurella multocida* Adhesion to Rabbit Respiratory Epithelium Using Lectins

**DOI:** 10.1155/2015/365428

**Published:** 2015-02-24

**Authors:** Magda Patricia Carrillo, Nhora María Martinez, María del Pilar Patiño, Carlos Arturo Iregui

**Affiliations:** Pathobiology Group, Laboratory of Veterinary Pathology, Faculty of Veterinary Medicine and Zootechnics, National University of Colombia, Bogotá D.C., Colombia

## Abstract

This study aimed to evaluate the ability of a panel of lectins to inhibit the ability of *Pasteurella multocida* to adhere to and affect the rabbit respiratory epithelium. Nasal septa from rabbit fetuses were cultured with various lectins before the addition of *P. multocida*. The percentage of bacteria adhering to the epithelium was evaluated semiquantitatively by indirect immunoperoxidase (IIP) staining. The goblet cells (GCs) were counted in semithin sections stained with toluidine blue and served as the main morphological criterion to evaluate the inhibitory effect of the lectins. The lectins PNA, WGA, RCA_120_, and DBA significantly inhibited the adhesion of *P. multocida* to the ciliated epithelium (*P* < 0.05) and prevented the pathogen-induced increase in the number of GCs (*P* < 0.05) compared with those of positive control tissues. In addition, VVA, SJA, UEA I, DSL, SBA, and ECL significantly inhibited the increase in GCs compared with that of the control tissues. The results suggest that less aggressive therapeutic strategies, such as treatment with lectins, may represent alternative approaches to control bacterial respiratory infections.

## 1. Introduction

Adhesion of pathogenic microorganisms to epithelial surfaces is an important step in the infection and colonization of a susceptible host [1–4]. Infection by pathogens is generally initiated by the specific recognition of host epithelial surfaces. Receptors present in the mucin layer can act as binding sites during microbial adhesion. Lectin/glycoconjugate interactions are known for their high specificity and play a significant role in the adhesion of bacteria and other microorganisms to the epithelial surfaces of their hosts. In their infection strategy, bacteria often use sugar-binding proteins, such as lectins and the adhesins pili or fimbriae, to recognize and bind to host glycoconjugates [5–7]. In gram-negative bacteria, the lipopolysaccharide (LPS) on the outer membrane has also been reported as an important structure involved in carbohydrate-lectin interactions [8–10].

Bacterial resistance to antibiotics is an ever-increasing concern. An alternative disease intervention strategy is to target important steps in disease pathogenesis rather than targeting the pathogen directly; in this case, we would seek to mitigate disease by limiting pathogen attachment to host cells and thereby reduce colonization using substances that, in contrast to antibacterial agents, do not destroy the pathogens but rather interfere with their first pathogenic step, namely, their attachment to host cells. Accordingly, antiadhesion therapies have been documented for the enteropathogen* E. coli* K99 in swine and calves; adhesion and infection of this bacterium can be prevented using carbohydrates (CHOs) [11–13]. In the same manner, the specific sialic acid adhesion of* Helicobacter pylori* to human gastric mucus and erythrocytes was inhibited by high molecular mass constituents derived from cranberries [14].

*Pasteurella multocida*, which is considered a normal component of the upper respiratory tract flora in a variety of animal species, is a well-known pathogen responsible for a range of diseases and economic losses in bovines, swine, canines, laboratory animals, rabbits, and birds [15–19]. This pathogen has also been associated with respiratory tract infections in humans [20]. Although the pathogenic process is not completely understood,* P. multocida* can transition from being a normal inhabitant of the host to a pathogen capable of causing disease and death. Strategies to control* P. multocida* disease include vaccines and antibiotics, which can have limited efficacy [21, 22].

A major capsular component of all* P. multocida* serogroup A strains is hyaluronic acid [23, 24], the adherent properties of which have been described [25, 26]; additionally, the LPS of* P. multocida* A serovar 3 strain Pm70 possesses CHO sequences similar to those found on several host epithelial surfaces [27, 28]. These findings suggest that both structures might be susceptible targets for antiadhesive therapy by their corresponding lectins or CHOs.

In a search for alternative strategies to control* P. multocida* infections with fewer side effects, this* ex vivo* study explored the possibility of inhibiting* P. multocida* adhesion to the respiratory epithelium (nasal septum) of fetal rabbits using lectins. The results showed that lectin pretreatment reduced the number of bacteria adhering to the apical surface of the epithelium and blocked the pathogen-induced increase in the number of GCs in respiratory tissues, thus raising the possibility of an alternative control strategy for bacterial infection.

## 2. Materials and Methods

### 2.1. *P. multocida* Strains

*P. multocida* isolates from Pm147/08 to Pm160/08 were obtained from the turbinates, trachea, or lungs of diseased rabbits with rhinitis and bronchopneumonia [29]. Routine microbiology tests and PCR amplification and sequencing of the hyaD gene in the cap locus, which encodes proteins involved in the synthesis and assembly of the type A capsule (GenBank accession number AF067175) [30, 31], confirmed the identity of* P. multocida* type A.

*P. multocida* isolates were passed through mice by intraperitoneal inoculation. Mice were euthanized after the first signs of disease, and the bacterium was recovered from the heart, liver, lung, and trachea and cultured on BHI agar at 37°C for 24 h before use. The bacterial mass was collected and diluted in glucose-enriched essential medium (MEM), achieving a final concentration of 10^7^ CFU/mL via counting and plating.

### 2.2. Lectins

A total of 18 distinct lectins from three commercial kits (Vector Laboratories) were used (Table 1). These lectins are extracted from various plants and included Con A, DBA, DSL, ECL, GSL I, GSL II, Jacalin, LCA, LEL, PNA, PSA, RCA_120_, SBA, SJA, STL, UEA I, VVA, and WGA. They were selected based on a range of specificities for multiple sugars [32–37].

### 2.3. *Ex Vivo* Culture of Rabbit Fetal Nasal Septa

This study was conducted with the approval of the Bioethics Committee of the Faculty of Veterinary Medicine of the National University of Colombia.

Eight pregnant female rabbits at their 26th gestational day were anesthetized with xylazine (5 mg kg^−1^) and ketamine (35 mg kg^−1^). The fetuses were delivered by caesarean and immediately euthanized by medullar sectioning; the females were also euthanized with an overdose of anesthetics immediately after the surgery. The skin, mandible, muscle, and palate of the fetuses were removed, and the nasal cavity was cross-sectioned with a sterile blade to obtain three slices, each 0.3 cm thick, from each animal. Sections were washed three times in MEM before tissue culture.

### 2.4. Inhibitory Effect of Natural Lectins on* P. multocida* Adherence to the Rabbit Respiratory Epithelium

To evaluate the potential inhibitory effects of lectins on* P. multocida* adhesion, an experiment was designed to block potential receptors for the bacterium on the apical membrane of epithelial cells. Six cross sections of nasal septa and bacteria were separately evaluated using each lectin (applied at 0.2 *μ*g mL^−1^); six additional sections cultured without* P. multocida* or lectin and three sections incubated with each lectin alone were used as negative controls; six additional explants were incubated only with the bacterium and were used as positive controls. Tissue sections were immersed in 10 mL MEM supplemented with lectin in a 5 cm diameter Petri dish and incubated in a humid chamber with 5% CO_2_ and 95% O_2_ for 1 h. The samples were then washed three times with MEM to eliminate the nonadherent lectin. Next, 10^7^ CFU of* P. multocida* was added to the samples, and they were incubated for 2 h. Three explants of each lectin treatment were fixed with 3.7% buffered formalin and three with Trump fixative (40% formalin, 25% glutaraldehyde) for 24 h. Control tissues were fixed in a similar manner [29, 38, 39].

### 2.5. Tissue Processing

#### 2.5.1. Immunohistochemistry

A polyclonal antiserum raised in an adult female sheep was used. Briefly, as a first dose, 250 *μ*g/mL of* P. multocida* was injected with complete Freund adjuvant (CFA); 7 days later, a booster dose with incomplete Freund adjuvant was applied. Finally, two doses of the antigen without adjuvant were inoculated one week apart. Animals were bled at 35 days p.i.; serum collected before inoculation served as a negative control. To eliminate cross-reactions, the serum was immunoadsorbed with normal nasal tissues of other fetuses. The serum was diluted 1 : 25 in sterile Tris-buffered saline (pH 7.6); 5 mL of the antiserum was then diluted in 1 mL of macerated sterile tissues previously washed with physiological salt solution. The mixture was centrifuged at 1000 rpm for 1 h at room temperature, and the supernatant was collected and frozen at −20°C until use. The working dilution of the primary antiserum was determined by indirect immunodot; a similar procedure was followed for the second antibody.

The number of bacteria adhered to the ciliated border of the respiratory epithelial cells was assessed by an indirect immunoperoxidase (IIP) technique [40, 41]. The tissues were embedded in paraffin and cut into 3 *μ*m thick sections. The polyclonal antiserum raised in sheep was used as the specific primary antibody, and a commercial antiserum against ovine IgG produced in donkey was used as a secondary antibody (Sigma, Aldrich) [42]. Nasal septa of rabbits affected by the rhinitic and pneumonic forms of the disease were used as positive controls for the IIP technique.

The level of* P. multocida* adherence to the epithelium was assessed by a semiquantitative procedure as follows: both epithelial surfaces of the nasal septa were considered the full area (100%) to which the bacteria could adhere, and a mean adherence level was determined; no bacteria adhering to the surface were scored as 0%; bacteria attached to >0–30% of the epithelial surface were considered to be of focal adhesion; bacteria adhered to >30–60% of the surface were considered to be multifocal; and bacteria adhered to >60% of the surface were interpreted to be of diffuse adherence.

### 2.6. Semithin Sections

Tissue sections fixed for 24 h in Trump solution were decalcified in 10% EDTA for seven days, washed with 0.1 mmol phosphate-buffered saline (pH 7.3), postfixed in 1% osmium tetroxide, dehydrated in an ascending alcohol gradient, and finally embedded in Epon 812 (Polysciences). Sections (0.5 *μ*m thick) were cut with a microtome (Microm) and stained with toluidine blue for approximately 30 s.

Tissue sections were evaluated by light microscopy using 100x objective. The number of cells (with cell nuclei used as the counting unit) in eight continuous fields of respiratory epithelium was analyzed in each of 3 replicates. The protective role of lectins was determined by calculating the percentage of GCs relative to those of other respiratory epithelial cells [29, 43]. In addition, increased GC activity was considered when cells showed an enlarged size, increased mucin release, and apical cytoplasm that protruded over their neighbor cells [44].

### 2.7. Statistical Analysis

The mean proportions of ciliated respiratory epithelium covered by adhered and nonadhered* P. multocida* were compared among the various lectin treatments and positive control tissues by ANOVA in a completely random model. To determine differences between treatments, a Dunnett test was performed, with *P* < 0.05 accepted as significant. To analyze the GC activity when the hypothesis was significant (*P* < 0.05), a Dunnett test was performed to compare each lectin treatment with the positive control [45].

## 3. Results

### 3.1. Inhibition of* P. multocida* Adhesion to the Respiratory Epithelium of Nasal Septa: IIP Staining

**Figure 1:**
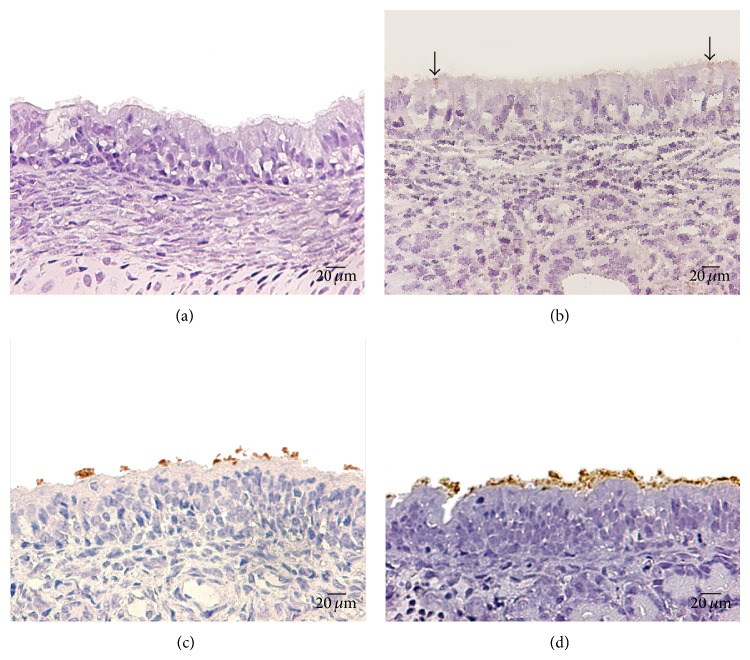
IIP technique for determining the percentage of the epithelial surface covered by adherent* P. multocida*. (a) Normal fetal rabbit respiratory nasal epithelium of IIP, negative control; light microscopy (LM), scale bar = 20 *μ*m. (b) Respiratory epithelium of the nasal septa of rabbit fetus preincubated with PNA plus* P. multocida* (sparse focal immunostaining (arrows); >0–30% of the epithelial surface); LM, scale bar = 20 *μ*m. (c) Tissue preincubated with VVA and* P. multocida* showing multifocal immunostaining (>30–60% of the surface covered with bacteria); LM, scale bar = 20 *μ*m. (d) Tissue incubated with* P. multocida* without lectins (positive control) showing generalized immunostaining (>60% of the epithelial surface covered by bacteria); LM, scale bar = 20 *μ*m.

Immunostaining of* P. multocida* on the apical surface of the respiratory epithelium in lectin-treated tissues ranged from localized (>0–30%) (Figure 1(b)) to multifocal (>30–60%) (Figure 1(c)); by contrast, in positive control tissues (tissues exposed only to the bacterium), the immunostaining was similar in all replicates, with a generalized granular appearance covering almost 90% of the epithelial surface (Figure 1(d)).

**Figure 2:**
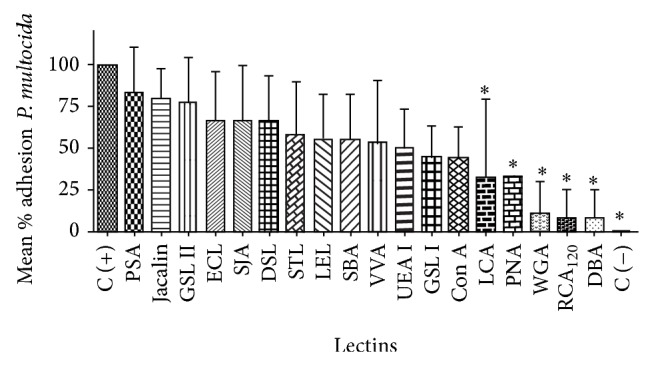
Inhibition of* P. multocida* adhesion to the ciliated respiratory epithelium of rabbit fetuses exposed to various lectins. Adhesion is expressed as mean percentage of the ciliated border epithelium coated by adhered bacteria. ^*^Significant inhibition (*P* < 0.05) compared with the positive control tissues. C (−): negative control.

The lectins LCA (*Lens culinaris*), PNA (*Arachis hypogaea*), WGA (*Triticum vulgaris*), RCA_120_ (*Ricinus communis*), and DBA (*Dolichos biflorus*) significantly (*P* < 0.05) inhibited* P. multocida* adherence to the ciliated border of respiratory epithelial cells compared with that of the positive control tissues (Figure 2).

### 3.2. Semithin Sections

**Figure 3:**
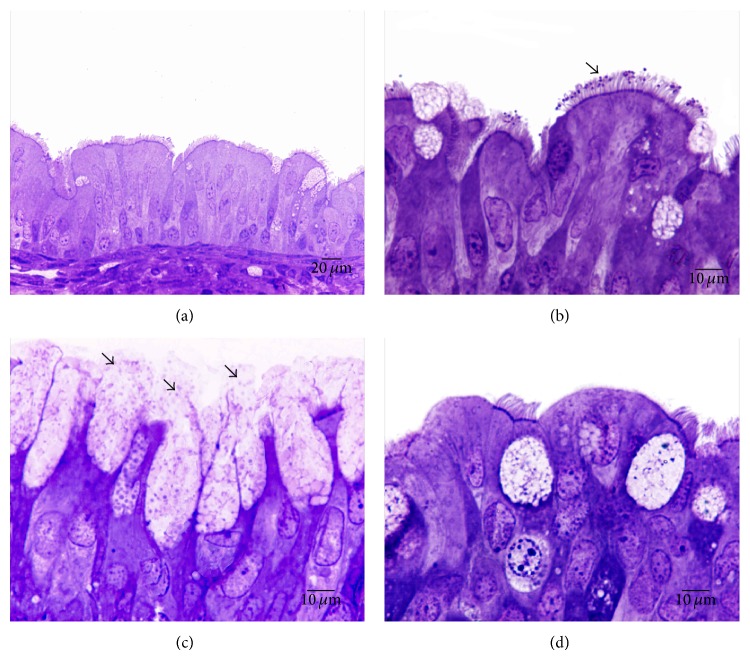
Toluidine blue staining of respiratory nasal epithelia of rabbit fetuses exposed to* P. multocida*. (a) Normal epithelium not exposed to bacteria or lectins; LM, scale bar = 20 *μ*m. (b-c) Nasal septa of positive control tissues exposed only to* P. multocida*. (b)* P. multocida* adhering to cilia (arrow); LM, scale bar = 10 *μ*m. (c) GCs showing increased numbers and excretory activity. All of the GCs are protruding above the apical limit of their neighboring cells and liberating their contents (arrows); LM, scale bar = 10 *μ*m. (d) Nasal epithelium exposed to both bacteria and PNA. The lectin inhibited the increase in the number of GCs, though some loss of cilia can still be observed; LM, scale bar = 10 *μ*m.

**Figure 4:**
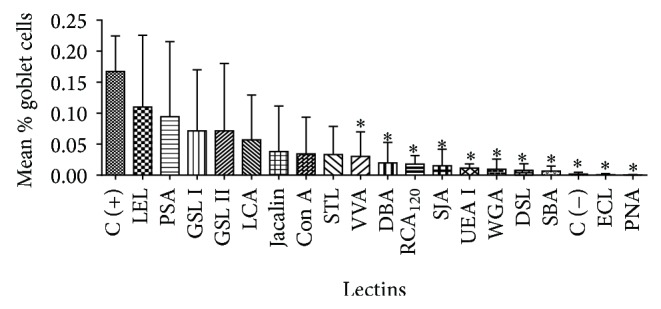
Inhibition of the increase in the percentage of GCs in the respiratory epithelia of nasal septa treated with various lectins. ^*^Significant inhibition (*P* < 0.05) by lectins compared with positive control tissues. C (−): negative control.

The respiratory epithelia of nasal septa exposed only to* P. multocida* showed an increase in the number of GCs compared with that of the control specimens (Figures 3(a) and 4). This increased number of GCs (Figure 3(c)) was accompanied by an enhanced production and release of mucus, such that the apical surface of these cells protruded above their neighboring ciliated cells, with their contents liberated into the lumen (Figure 3(c)). No increase in the number or activity of GCs was observed when nasal septa were incubated separately with each lectin.

The lectins VVA (*Vicia villosa*), DBA (*Dolichos biflorus*), RCA_120_ (*Ricinus communis*), SJA (*Sophora japonica*), UEA I (*Ulex europaeus I*), WGA (*Triticum vulgaris*), DSL (*Datura stramonium*), SBA (*Glycine max*), ECL (*Erythrina cristagalli*), and PNA (*Arachis hypogaea*) also showed significant inhibitions (*P* < 0.05) of the GC number and activity (Figure 4).

## 4. Discussion

The current strategies for preventing* P. multocida* infection in susceptible species, including rabbits, are limited to a few vaccines and antibiotics, but these approaches have shown inconsistent results [46–49]. In this study, we have explored a novel strategy to impede the adherence of this microorganism to the respiratory epithelia of rabbits using lectins while simultaneously investigating which molecules could protect the epithelium without having to kill the bacteria. Implementing an* ex vivo* experimental protocol, our data demonstrate that certain lectins inhibited the attachment of* P. multocida* to the respiratory epithelia of fetal rabbit nasal septa and also prevented the pathogen-induced increase in the number of GCs.

In this study, treatment with the lectins PNA (Gal *β*1–3/GalNAc), WGA (GlcNAc/Neu5Ac), RCA_120_ (Gal*β*1–4/GalNAc), DBA (GalNAc (*α*1–3)/GalNAc), and LCA (Man/Glc) significantly inhibited* P. multocida* adhesion to rabbit nasal respiratory epithelia and, in the cases of PNA, WGA, RCA_120_, and DBA also prevented an increase in the number and activity of GCs. These results suggest three possible explanations for the inhibitory activity of these lectins: (1) LCA, PNA, WGA, RCA_120_, and DBA bind to their specific CHO moieties present on the apical surface of the respiratory epithelium, thereby masking putative CHO receptors for corresponding lectins on the bacterial surface similar to those targeted in this work; (2) these lectins, on their side, recognize CHOs on the bacterial capsule and/or its LPS that serve as ligands for the corresponding lectin receptors on the apical membrane of the respiratory epithelium; and (3) both events occur simultaneously. Mason et al. [50] demonstrated that the PNA, LCA, RCA_120_, and WGA lectins recognize CHOs on the upper respiratory epithelium of laboratory rodents, partially confirming our results. In addition, Perfumo et al. [51] reported that lectins similar to those tested in this study (DBA, SBA, PNA, and RCA_120_) bind to receptors on the respiratory epithelial cells of the nasal cavity of healthy swine, as well as those suffering from atrophic rhinitis caused by* P. multocida* and* B. bronchiseptica*. Taken together, these findings indicate that CHOs and lectins with corresponding specificities are present on the apical surfaces of the respiratory epithelia of various animal species and most likely also on the surfaces of* P. multocida* and* B. bronchiseptica*, serving as receptors and ligands, respectively.

WGA recognizes GlcNAc (*β*1) and D-glucuronic acid residues, which are constituents of hyaluronan, a major nonsulfated glycosaminoglycan ubiquitous in all connective tissues; hyaluronan has also been described on both the apical and basal surfaces of the airway epithelium [52–55] and in tracheal secretions [56]. Hyaluronic acid is also a major capsular component of all* P. multocida* serogroup A strains [23, 24].* P. multocida* serogroup A has been shown to adhere strongly to HeLa cells, turkey air sac macrophages, and alveolar macrophages; bacterial adhesion was reduced by treatment of the bacteria with hyaluronidase (to reduce the amount of capsule) or the addition of hyaluronic acid [25, 26]. Similarly, a spontaneous acapsular variant of* P. multocida* serogroup A did not adhere to turkey air sac macrophages [26]. Glorioso et al. [57] studied the adhesion of* P. multocida* isolated from rabbits to monolayers of HeLa cell cultures and to parakeratotic pharyngeal cells. Their most significant finding was that GlcNAc treatment inhibited bacterial adhesion to both cell lines. These results suggest that lectin-like molecules that serve as receptors for the microorganism are present on both epithelial surfaces. The main adherent role was attributed to* P. multocida* fimbriae [58]. These findings were complemented by those of Al-Haddawi et al. [59], who demonstrated that* P. multocida* A3 fimbriae isolated from rabbits recognize GlcNAc on the cilia of the respiratory epithelium; they were also able to competitively block this binding using the same CHO. Conversely, Jacques et al. [60] and Hatfaludi et al. [8] state that, despite the fact that* P. multocida* possesses various structures able to contribute to its binding of the respiratory epithelial cells of swine and rabbits* in vitro*, the LPS and type IV fimbria are likely the main mediators of the adhesion, acting as the CHO and lectin ligands, respectively. It is conceivable that, in our studies, WGA simultaneously bound hyaluronan residues on the apical surface of the respiratory epithelium and on the capsule of* P. multocida*, thereby exerting a double inhibitory effect.

However, the protective effect of WGA in this research could also be attributed to the inhibition of adherence of* P. multocida* LPS to hyaluronan on the apical membrane of the nasal respiratory epithelial cells. Protective activity against LPS-induced septic shock, acute lung injury, and airway hyperreactivity has been demonstrated by pretreatment with high molecular weight hyaluronan (<5 × 10^5^), indicating that soluble hyaluronan can prevent the adherence of LPS to membrane-bound hyaluronan [61, 62].

The increase in the number and activity of GCs observed in this study could be due to the stimulation of the bacterium itself but is more likely related to the presence of substances liberated by* P. multocida*, mainly its LPS. Using the same* ex vivo* model as in this study, a previous study from our group exposed nasal cultures to* P. multocida* or its LPS separately; in both cases, the main response of the respiratory epithelium was a statistically significant increase in the number and activity of GCs compared with those of controls [29]. Previous studies have also reported hyperplasia and increased activity in the GCs of the respiratory epithelium following exposure to LPS [63–65]. Intranasal instillation of endotoxin in rats induces an inflammatory response characterized by an increase in the quantity or secretion of mucosubstances, as well as the infiltration of inflammatory cells into the epithelium, mainly PMNs [63]. Several mechanisms have been proposed to explain the positive effect of LPS on mucus production. Aside from the activity of various components of the PMNs, the expression of mucin genes, without the need for other secondary mediators, has been induced in cell cultures and nasal explants exposed only to endotoxin [66, 67]. Recent* in vivo* and* in vitro* work on the LPS of* Pseudomonas aeruginosa* reinforced these observations, as the expression of the mucin MUC5AC in mucin-producing cells of the airways was stimulated by treatment with this molecule [68]. Additional experiments by our group, in which the nasal septa of rabbit fetuses were only exposed to* P. multocida*, showed that the LPS was spontaneously released by the bacterium on the apical surface of the epithelial cells, where it entered the ciliated cells. This event was accompanied by increases in the number and activity of GCs, without any evidence of inflammatory cells [69]. These results reinforce the hypothesis that the LPS of* P. multocida* was one of the main targets of the inhibitory effect of the lectin WGA observed in this work.

Except for LCA, all lectins that prevented* P. multocida* adherence to the epithelium (PNA, WGA, RCA_120_, and DBA) also inhibited the increase in the number of GCs. It is not clear why LCA did not also have an inhibitory effect on these cells.

A different effect was found for VVA, SJA, UEA, DSL, SBA, and ECL, which significantly prevented the elevation in the number of GCs but did not significantly inhibit* P. multocida* adherence to the respiratory epithelium. Although we do not have a convincing explanation for the lack of inhibitory effect on* P. multocida* adhesion, part of the inhibitory effect of VVA, SJA, SBA, and ECL on GC number could be attributable to their specificity for CHOs residues containing Gal/GalNAc (and of DSL for GlcNAc), as these are important constituents of the LPS of* P. multocida* serovar 3, Pm70 [27].

Analysis of the* P. multocida* 3 Pm70 genome sequence identified two predicted filamentous hemagglutinin genes (fhaB1 and fhaB2) that might have similar adherent activities as that of the filamentous hemagglutinin protein of* Bordetella pertussis*, FhaB-FhaB1 FhaB2, for the adhesion to host cells [8, 70, 71]. This protein has at least three separate binding activities: a glycosaminoglycan binding site [72–74], an integrin-binding arginine-glycine-aspartate (RGD) sequence [75, 76], and a CHO recognition domain that mediates attachment to ciliated respiratory epithelial cells and macrophages [77–80]. The FhaB1 and FhaB2 proteins of* P. multocida* could have similar ligand affinity for CHO receptors on the airway epithelia of rabbits.

The results of this study indicate that* P. multocida* type A binds to the apical surface of rabbit nasal respiratory epithelia through several CHO-containing receptors, likely via bacterial surface structures, such as the capsule, LPS, type IV fimbriae, or lectin-like structures that may specifically bind to those glycosidic receptors, all of which would be potential targets for the inhibitory effect of the lectins observed in this work. We propose that, in addition to the potential use of lectins to inhibit the deleterious effects of* P. multocida* adhesion and colonization, it could also be more effective to employ the corresponding sugars, that is, D-Man, D-Glc, and GlcNAc, the effects of which have recently been demonstrated [81]. This approach would allow the key receptors on the respiratory epithelial cells to be occupied by the sugars or endocytosed upon sugar binding, reducing their numbers and leaving no binding sites for the microorganisms' sugar molecules [9, 13, 82]. The use of natural substances to inhibit pathogen adhesion to host cells might be an advantageous strategy that does not exert evolutionary pressure to generate more pathogenic forms of the microorganisms [6, 9].

## 5. Conclusions

A number of lectins were found to impede the attachment of* P. multocida* to the respiratory epithelia of fetal rabbit nasal septa* ex vivo*. The inhibition of* P. multocida* adhesion protected the respiratory cells from the lesions caused by the pathogen, and it is therefore highly probable that the undamaged mucosa would be able to clear the pathogen by innate protective mechanisms, such as mucociliary clearance. Preventing* P. multocida* colonization and cell damage in the upper respiratory tract may impede the bacteria from reaching deeper regions of the lungs. Our results support future investigations into whether the CHOs identified herein can also inhibit* P. multocida* adhesion in rabbits* in vivo*.

## Figures and Tables

**Table 1:** Lectins and their characteristics [34, 35, 37]. Man, mannose; Glc, glucose; GalNAc, N-acetylgalactosamine; GlcNAc, N-acetylglucosamine; Gal, galactose; Fuc, fucose; Neu5Ac, N-acetylneuraminic acid.

Source of lectin			
Abbreviation	Latin name	Common name	Carbohydrate specificity
Con A	*Canavalia ensiformis *	Jack bean	Man/Glc
DBA	*Dolichos biflorus *	Horse gram	GalNAc(*α*1-3)/GalNAc
DSL	*Datura stramonium *	Jimson weed or thorn apple	(GlcNAc)2-4
ECL	*Erythrina cristagalli *	Coral tree	Gal/GalNAc
GSL I	*Griffonia simplicifolia I *	Unknown	Gal/GalNAc
GSL II	*Griffonia simplicifolia II *	Unknown	GlcNAc
Jacalin	*Artocarpus integrifolia *	Jackfruit	Gal/GalNAc
LCA	*Lens culinaris *	Lentil	Man/Glc
LEL	*Lycopersicon esculentum *	Tomato	(GlcNAc)2-4
PNA	*Arachis hypogaea *	Peanut	Gal *β*1-3/GalNAc
PSA	*Pisum sativum *	Garden pea	Man/Glc
RCA_120_	*Ricinus communis *	Castor oil bean	Gal*β*1-4/GalNAc
SBA	*Glycine max *	Soybean	Gal/GalNAc
SJA	*Sophora japonica *	Japanese pagoda tree	Gal/GalNAc
STL	*Solanum tuberosum *	Potato	(GlcNAc)2-4
UEA I	*Ulex europaeus I *	Gorse or furze	Fuc
VVA	*Vicia villosa *	Hair vetch	GalNAc
WGA	*Triticum vulgaris *	Wheat germ	GlcNAc/Neu5Ac
